# Comparison of bone mineral density between female amateur triathletes and nonathletes: A cross-sectional study

**DOI:** 10.1177/00368504241261844

**Published:** 2024-07-25

**Authors:** Guilherme Cruz Correa Netto Soares, José Geraldo Gomes Barbosa Junior, Aldo Seffrin, Lavínia Vivan, João Victor Rosa de Freitas, Gustavo De Conti Teixeita Costa, Claudio André Barbosa B de Lira, Rodrigo Luiz Vancini, Katja Weiss, Beat Knechtle, Marilia Santos Andrade

**Affiliations:** 1 Sports Medicine Residency Program, Department of Orthopedics and Traumatology, 28105 Federal University of São Paulo, Brazil; 2 Department of Physiology, 28105 Federal University of São Paulo, Brazil; 3 Human and Exercise Physiology Division, Faculty of Physical Education and Dance, 67824 Federal University of Goiás, Brazil; 4 Center for Physical Education and Sports, 28126 Federal University of Espírito Santo, Brazil; 5 Institute of Primary Care, University of Zurich, Zurich, Switzerland; 6 Medbase St Gallen Am Vadianplatz, St Gallen, Switzerland

**Keywords:** Bone mass, triathlon, body composition, women, X-ray absorptiometry

## Abstract

**Purpose:**

Physical inactivity is considered an important risk factor for osteoporosis, however, some athletes performing extremely high training volumes can also develop bone mass loss. Moreover, the effect of total body mass or body surface area on bone mineral density remains controversial. Therefore, the aim of this study was to compare the absolute bone mineral density and bone mineral density adjusted to body surface area between amateur triathletes and nonactive women.

**Methods:**

Forty-two healthy women (23 amateur triathletes and 19 nonactive individuals) were evaluated for body composition using a dual-energy X-ray absorptiometry system.

**Results:**

Compared to nonactive women, amateur triathletes exhibited lower body mass index (*p *< 0.001), lower bone mineral density (*p *< 0.001), and body surface area (*p *< 0.001). However, bone mineral density adjusted by body surface area in the triathletes was higher than in the nonactive women (*p *= 0.03).

**Conclusion:**

These findings showed that amateur triathles presented lower absolute bone mineral density, but higher bone mineral density adjusted to body surface area. Future studies are recommended to identify if the higher bone mineral density adjusted to body surface area are associated with a lower bone fragility.

## Introduction

Physical inactivity is considered an important modifiable risk factor for osteoporosis.^1^ Despite the well-established benefits of physical activity, 30% of the world population remains inactive.^2^ The ideal amount of exercise to gain or maintain bone mass has yet to be determined. Current evidence suggests that bone gain depends on the exercise modality, volume, and intensity^3–5^ For overall health benefits in the general population, the American College of Sports Medicine (ACSM) recommends at least 60 min sessions of strength (2–3 times a week) and aerobic training (2–3 times a week),^6^ or an average of 5 h of physical activity per week. However, when the training volume exceeds ACSM recommendations, an energy imbalance between dietary calories and energy expenditure can occur, resulting in a syndrome called Relative Energy Deficiency in Sport (RED-S). Paradoxically, RED-S is characterized by changes in bone mineral density (BMD) and increases the risk of fracture.^7^

Excess fat mass can also significantly impact bone mass. For many years, it has been thought that overweight and obesity can be protective factors against osteoporosis in adults.^8,9^ More recently, this relationship has become controversial and has been contested in the literature.^10,11^ It was previously accepted that women with greater body mass have greater BMD as an adaptive mechanism to support a greater load on the skeletal system.^12^ However, recent evidence has suggested that a greater body mass may exceed the ability of the bones to support the body, implying a greater risk of bone fracture in this population. For example, a previous study demonstrated that the risk of ankle fracture is greater by 60% in obese women than in nonobese women^13^ despite a recent systematic review and meta-analysis showing that BMD is higher in subjects with obesity.^13^

A variable that can potentially be factored in the evaluation of bone mass is total body surface area (BSA). The BMD adjusted to BSA may offer additional and useful information about the bone capacity to support the mechanical load of the body. Furthermore, it is critical to determine whether it is necessary to compare the bone mass among individuals with different body compositions, such as athletes and inactive individuals. Thus, a study to clarify this question is warranted.

Therefore, the aim of the present study was to compare the absolute BMD and BMD adjusted to BSA between triathletes, who train at levels above the ACSM recommendation, and healthy inactive women with physical activity below ACSM recommendations. This study tested the hypothesis that triathletes have lower absolute BMD but higher BMD adjusted to BSA than nonactive women due to the high amount of physical exercise.

## Materials and methods

### Ethical approval

The protocol of this study was approved by the Human Research Ethics Committee of the Federal University of São Paulo, Brazil (Approval number: 5.059.538, October 25, 2021) and conformed to the principles of the Declaration of Helsinki. Each participant was informed of the aims, benefits, and risks of the study and signed a consent form before participating.

### Participants

Two groups of healthy women were recruited in the study: Amateur triathletes (Group 1) and nonactive women (Group 2). Women in Group 1 were amateur triathletes recruited directly or via social media. Participants in Group 2 were nonactive women recruited on social media. The sample size was calculated using G*Power version 3.1.9.2 (Franz, Universität Kiel, Germany). For power level calculation, a t-test family was selected, and the mean, standard deviation, and effect size (Cohen d) were included in the calculation. Using BMD data from a pilot study (n = 4), which were 1.15 ± 0.06 g/cm^2^ for athletes and 1.19 ± 0.03 g/cm^2^ for controls, each group should have a minimum of nineteen participants in each to detect a difference with 80% power and significance level of 5%. A total of 42 women participated, 19 individuals in Group 1 and 23 individuals in Group 2.

The inclusion criterion for Group 1 was training for at least 3 years at volumes or intensities exceeding ACSM recommendations. The inclusion criterion for Group 2 was women with physical activity below the hours recommended by the ACSM. Participants were asked if they performed at least 60 min sessions of strength (2–3 times a week) and aerobic training (2–3 times a week) or an average of 5 h of physical activity per week.^6^ Exclusion criteria included the use of any drug or hormone affecting bone mass, diseases affecting bone metabolism, and pregnancy. Individuals, particularly for Group 1, were excluded if they had signs or symptoms of Female Athlete Triad. The Low Energy Availability in Females Questionnaire (LEAF-Q), which is an instrument validated for Portuguese and widely used to identify female athletes at risk for the Female Athlete Triad, was applied.^14^

### Study design

This was a cross-sectional study conducted at the Olympic Training and Research Center in São Paulo, Brazil. Data collection were performed from February to May 2023. Both amateur triathletes and nonactive women visited the laboratory once for anthropometric measurements and evaluation of body composition. Participants also responded to the following open-ended questions: How many hours per week do you train running? How many hours per week do you train cycling? How many hours per week do you train swimming? How many hours per week do you do strengthening exercises? Do you have any chronic disease? If yes, which one? Do you take any drugs for continuously?

### Experimental procedures

#### Body composition and BMD measurement

Height and body mass were measured using a calibrated stadiometer and body mass balance, respectively, with corresponding precisions of 0.1 cm and 0.1 kg. Body mass index (BMI) was calculated. A dual-energy X-ray absorptiometry system (DXA software version 12.3, Lunar DPX, GE Healthcare, Wisconsin, USA) was used to determine body composition and BMD. DXA is considered the gold standard method for assessing these measures.^15^ Prior to all examinations, the device was calibrated according to the manufacturer's recommendations. Although DXA involves exposure of patients to radiation, it is considered very safe due to the minimal dosage used.^16^ DXA scans were performed following a previously developed protocol. A qualified professional who was experienced with the method performed the DXA scan. All women were evaluated in the supine position wearing comfortable clothes and no accessories. BMD and bone mineral content (BMC) of the whole body were measured, and the results were presented in g/cm^2^ for analysis.

### Statistical analysis

Data were expressed as mean and standard deviation. Data normality and homogeneity were tested using the Shapiro–Wilk and Levene tests, respectively. An unpaired *t*-test was used to compare anthropometric data and bone density parameters of triathletes and nonactive women. The effect size for differences between groups was determined by dividing the mean difference between the two groups by the pooled standard deviation. The effect sizes were used to determine the magnitude of any change and were classified into the following: *d* < 0.2: no effect; 0.2 ≤ *d* < 0.5: small effect size; 0.5 ≤ *d* < 0 .8: medium effect size; and *d* ≥ 0.8: large effect size.^17^ The level of significance was set at *p *< 0.05. Statistical analyses and tests were performed using IBM SPSS Statistics (version 26, IBM SPSS, Chicago, IL, USA).

## Results

The female amateur athletes train 13.2 ± 3.0 h per week (minimum of 8 h and maximum of 18 h per week). The age, height, body mass, and BMI of the participants are presented in Table 1. Age and height did not significantly differ between groups. However, amateur triathletes had lower body mass (*p *< 0.001) and lower BMI (*p *< 0.001) than nonactive women. Regarding body composition, amateur athletes presented higher lean mass (*p* = 0.018) and lower fat mass (*p* < 0.001) than nonactive women.

**Table 1.** Participant characteristics.

Variables	Group 1 (triathletes) (n = 23)	Group 2 (nonactive) (n = 19)	*p*-value	Effect Size (*d*)	CI (95%)	Power (1−β)
Age (years)	42.1 ± 8.2	45.9 ± 4.1	0.08	0.6	0.4–0.9	0.69
Height (cm)	162.4 ± 5.9	164.0 ± 6.6	0.425	0.25	0.01–4.7	0.73
Body mass (kg)	57.6 ± 6.3	70.6 ± 8.9	< 0.001	1.80	1.4–2.2	0.94
BMI (kg/m^2^)	21.8 ± 1.9	26.2 ± 2.8	< 0.001	1.83	1.4–2.4	0.95
Lean mass (kg)	41.8 ± 6.4	37.6 ± 3.9	0.018	0.79	0.7–0.9	0.65
Fat mass (%)	13.1 ± 7.0	28.6 ± 6.3	< 0.001	2.32	1.7–3.2	0.99

Data are presented as mean ± standard deviation. BMI: body mass index; CI: confidence interval.

Both BMD (*p *= 0.006) and BSA (*p *< 0.001) were significantly lower in Group 1 than in Group 2. However, BMD adjusted by BSA in the triathletes was higher than in nonactive women (*p *= 0.03) (Table 2). Regarding the bone mineral content (BMC) there were no significant differences (*p *= 0.05) between the two groups.

**Table 2.** Comparison of BMD, BSA, and BMD/BSA between groups.

Variables	Group 1 (triathletes) (n = 19)	Group 2 (nonactive) (n = 19)	*p*-value	Effect Size (*d*)	CI (95%)	Power (1-β)
Absolute BMD (g/cm^2^)	1.17 ± 0.05	1.24 ± 0.08	0.006	1.05	1.0–1.1	0.77
BSA (m^2^)	1.61 ± 0.10	1.79 ± 0.13	< 0.001	1.55	1.3–1.9	0.94
BMD/BSA	0.73 ± 0.06	0.69 ± 0.04	0.03	0.78	0.6–0.9	0.72
BMC (kg)	2.49 ± 0.24	2.70 ± 0.42	0.05	0.61	0.4–0.9	0.58

Data are presented as mean ± standard deviation. BMD: bone mineral density; BSA: body surface area; BMC: bone mineral content; CI: confidence interval.

## Discussion

The main results of the present study were: (i) triathletes had lower body mass and lower absolute BMD than nonactive women; (ii) triathletes had lower BSA than nonactive women; (iii) triathletes exhibited higher BMD adjusted to BSA than nonactive women. These results confirm the initial hypothesis of the present study.

The present study showed that female amateur athletes presented training characteristics very similar (hours per week) as previously reported to amateur triathles (13 h/week)^18^; however, lower than elite triathles (20 h/week).^19^ Regards to bone status, absolute BMD for triathletes was lower than for nonactive women. This lower absolute BMD could be interpreted as a negative consequence of high training volume in these athletes. Among preventive measures against osteoporosis, physical activity has been proven to be fundamental for bone mass accumulation. In contrast, several studies have shown that very high volumes of physical exercise, when associated with low caloric intake, can lead to a negative energetic balance. This can impair several physiological functions, including bone acquisition and bone health.^20^ In this scenario, athletes may not benefit from the positive effects of physical exercise on bone mass and may even have bone masses lower than sedentary controls.^21,22^ In apparent agreement with this line of thought, the data from the present study show that the absolute BMD is lower in triathletes than in controls. However, it is mandatory to highlight that previously published data for absolute BMD for a population of South American women of the same age were 1.12 ± 0.10 g/cm^2^,^23^ evidencing that the present data for triathletes were very similar, or even higher than the reference data from literature. Therefore, this lower absolute BMD in triathletes than in controls of the present study should be interpreted with caution because this group also had very different body composition and body mass, which may affect the comparison of absolute BMD values.

The importance of body composition can be highlighted since nonactive women showed higher absolute BMD measurements, but this cannot rule out the possibility that the bone is still insufficient to resist larger forces, such as when a heavier person falls or when exposed to various biomechanical stressors.^11^ Therefore, higher absolute BMD may not always reflect a lower risk of fractures. Consistent with this, a study from a fracture liaison service in the United Kingdom in 2011 reported normal absolute BMD, measured by DXA, in obese postmenopausal women with fragility fractures.^24^

In the same way that high absolute BMD values in obese individuals may not be enough to protect them from fragility fractures, it is also possible to hypothesize that low absolute BMD values presented by athletes, presenting a low percentage of fat mass and high lean mass, may not necessarily indicate a greater stress fracture risk.

Assessing BMD adjusted to BSA may be a feasible alternative to compare the bone mass among women with different body compositions and anthropometric dimensions. This variable may reflect bone health more reliably, but this hypothesis needs to be confirmed by future studies. Moreover, the data on BMD adjusted to BSA may indicate that triathletes have a more optimized BMD than nonactive women. These findings are in line with previous reports in the literature showing that obese women have a higher risk of ankle, leg, and humeral fractures.^13,25^ In these cases, a threshold at which bone mass cannot support the body mass may have been surpassed, leading to an increased risk of fracture.

Notwithstanding, obese women have a lower risk of hip fractures.^13,25^ The underlying reasons for this site-specific association are not yet fully understood. It has been suggested that soft tissue padding in the pelvic area allows energy dissipation after trauma or a fall, contributing to the protective effect of obesity against hip fractures.^24^

An important strength of the study is the use of a reliable and valid instrument, DXA, to measure BMD. However, the study has some limitations. First, the cross-sectional design does not support causality. Therefore, prospective research should be conducted to determine the long-term consequences of triathlon training on BMD. A second limitation is the lack of nutritional status evaluation. Third, the descriptive data about the athletes’ weekly training volume was assessed using a non-validated questionnaire and there may be some memory bias regarding this descriptive aspect of the study participants. Finally, the BSA determined in this study was estimated through the Mosteller formula^26^ rather than actual measurement.

## Conclusion

The present results showed that absolute BMD is lower and BMD adjuted by BSA is higher among amateur athletes than among healthy controls. As the athletes presented higher lean mass, despite the lower body mass, future reseaches are recommended to investigate if the lower absolute BMD are associated with bone fragility among amateur triathletes, or if the BMD adjusted to BSA may be an alternative measured to evaluate bone fragility among them.
